# Risk Factors for Revolving Door in Children and Adolescents with Psychiatric Disorders

**DOI:** 10.3390/jcm10215004

**Published:** 2021-10-27

**Authors:** Barbara D’Aiello, Deny Menghini, Roberto Averna, Milena Labonia, Stefano Vicari

**Affiliations:** 1 Child and Adolescent Neuropsychiatry Unit, Department of Neuroscience, Bambino Gesù Children’s Hospital, IRCCS, 00146 Rome, Italy; barbara.daiello@opbg.net (B.D.); deny.menghini@opbg.net (D.M.); roberto.averna@opbg.net (R.A.); milena.labonia@opbg.net (M.L.); 2 Department of Human Science, LUMSA University, 00193 Rome, Italy; 3 Department of Life Science and Public Health, Università Cattolica del Sacro Cuore, 00168 Rome, Italy

**Keywords:** psychiatric readmissions, psychiatric rehospitalizations, self-injurious, suicidal ideation, behavioural and emotional symptoms, dysregulated profile

## Abstract

Revolving Door (RD) is a frequent phenomenon afflicting children and adolescents with psychiatric diagnoses. Nevertheless, risk factors for RD are still a matter of debate. To better understand RD phenomenon, we conducted a retrospective study on 224 children and adolescents (165 females and 59 males, aged 6–16 years) with a psychiatric hospitalization, taking the multiple risk factors together. At this aim, 108 patients with multiple hospitalizations and 116 patients with only one hospitalization were compared on demographic characteristics, clinical conditions, psychiatric ward stay, and post-discharge management factors. More than half of psychiatric patients were readmitted within three months of discharge. RD patients presented greater severity of illness, needed longer stays, and were more frequently placed in residential facilities than non-RD patients. Non-suicidal self-injurious and adoption were the main predictors of RD. Clinical instruments that detected behavioural and emotional symptoms, suicidal ideation severity, and level of impairment of the person’s functioning were useful to identify patients at high risk for RD. In conclusion, our findings pointed out that several risk factors have to be considered to better understand and, in the future, prevent RD phenomenon.

## 1. Introduction

The term “revolving door” (RD) indicates repeated hospitalizations of the same patient in psychiatric units [1]. Psychiatric hospitalizations represent a huge burden in terms of costs and stress [2].

According to follow-up studies, 21–26% of adolescents in psychiatric care become frequent users of psychiatric services [3], and about a quarter of discharged patients experienced readmissions within one year [4,5,6]. The highest risk for rehospitalizations occurs within the first 3 months from discharge [4], and the first hospitalization length correlates with the risk of readmission [7,8,9]. Indeed, each additional day of hospitalization, during the first episode of psychiatric hospital stay, increases the possibility of rehospitalizations by 17% [10]. 

Findings showed that the most consistent predictors for rehospitalizations are the severity of symptoms and the global functioning of patients [2,5,11]. Mixed evidence exists both for and against the role of diagnosis in predicting RD. Some studies concluded that psychiatric diagnoses do not appear to predict the rehospitalizations [2,8,10,12,13,14] while other evidence showed that adolescents with psychotic disorder [15,16,17,18], schizophrenic disorder [19,20] and bipolar disorder [6,16,17,18] are at high risk for the rehospitalizations. Moreover, the exacerbation of psychiatric symptoms is considered responsible for the rehospitalizations (in 73–85% of the cases) within 30 days after discharge [8] and the presence of one or multiple psychiatric comorbidities associated with an increased likelihood of single and multiple readmissions [6].

Among the risk factors for rehospitalizations, clinical conditions such as suicidal ideation, self-injurious behaviours, and suicide attempts have the highest prevalence [9,21,22]. Indeed, adolescents with repeated rehospitalizations are twice likely to have suicide attempts [23,24]. In particular: 18% of them attempted suicide within 6 months from discharge, and 28% are readmitted to psychiatric units because of suicide risk [25]. Furthermore, suicidal ideation [26] and the presence of self-injurious thoughts and behaviours after discharge are found to be strongly related to rehospitalizations [14,25,26,27,28,29]. 

Additionally, psychiatric ward readmissions have been linked to insufficiently adequate services and treatments [30]. The quality of post-discharge services has been identified as an important factor in preventing hospitalizations [10,19,31,32]. Discharged adolescents with lower levels of care showed greater risks for the rehospitalizations than patients receiving higher levels [5], and receipt of supportive services reduced risk by 76% compared to not receiving any post-discharge services [10].

Demographic characteristics, such as age, gender, familial psychiatric history, and socioeconomic status, have also been suggested as risk factors for rehospitalizations. Some studies documented that younger adolescents who received the first hospitalization at an early age have been more likely to have more hospitalizations across the lifespan than older ones [10,15]. Contrarily, other studies found that older adolescents have been more often readmitted to a psychiatric ward than younger ones [2,5,16,19]. Concerning gender, females are more often readmitted to the hospital than males [11,33], with some exceptions [14,26,32]. The presence of a family history of mental health issues is a further risk factor for rehospitalizations [24,34,35]. Similarly, conflicting or disengaged child-parent relationships [4] as well as adverse environmental and social factors [36] may negatively affect child mental health and, thus, increase the risk of readmission to psychiatric care [24]. Specifically, parental stress and illness and a mono-parental family have been identified as the proximal cause of 13.4% of readmissions [8]. Children exposed to severe childhood victimization or youth living in foster care have also been more likely to be re-hospitalized [37].

Some clinical instruments have proved useful in detecting patients at high risk for rehospitalizations. In particular, the Child Behaviour Checklist questionnaire [38] was identified as a helpful tool in patients with a mood disorder, showing that internalizing profiles predicted suicide attempts and rehospitalization at 3 and 12 months [21]. Moreover, the dysregulated profile of CBCL, signifying simultaneous clinical scores on anxious/depressed, attention problems, and aggressive behaviour subscales, has been related to suicide attempts, ideation suicide [39,40,41,42], and psychiatric hospitalizations [21]. Similarly, the Columbia-Suicide Severity Rating Scale [43] has been adopted to investigate suicide attempts in adolescents at risk of hospitalization [2,9]. Indicator of functional impairment as the Global Assessment of Functioning Scale has been detected as a possible predictor for rehospitalization [4,5,15]. However, these previous studies have mainly highlighted the association between rehospitalizations and specific psychiatric diagnosis, as a mood disorder, or specific conditions as suicide attempts or emotional dysregulation. 

To our knowledge, clinical instruments have not been already employed to identify whether specific behavioural and emotional symptoms predicted rehospitalizations and different factors affecting readmissions, such as clinical conditions, demographic characteristics, and psychiatric ward stay, are still a matter of debate and not investigated together in the same study.

The current study was aimed at better understanding RD phenomenon taking together several risk factors for the rehospitalizations. In particular, it was investigated the role of clinical conditions, such as the psychiatric diagnosis, psychiatric comorbidities, the presence of self-injurious attempt behaviours (non-suicidal self-injurious and suicidal attempts); psychiatric ward stay, such as the number of admissions, the time elapsed between admissions, length of stay, and post-discharge management; demographic characteristics, such as age, gender, IQ, familial history of psychiatric disorders and adoption.

A secondary aim was to identify clinical instruments useful to detect symptoms associated with high risk for repeated rehospitalizations. Screening tools for assessing global functioning, behavioural and emotional symptoms, and suicidal risk may help clinicians to find the most appropriate project at the discharge.

## 2. Materials and Methods

### 2.1. Participants

We retrospectively selected inpatients with one or more hospitalizations per year over 3 years (from January 2017 to December 2019) at a Child and Adolescents Neuropsychiatry Unit ward that hosts children and adolescents for a maximum of 8 beds a day.

We included only children and adolescents hospitalized for the first time over the 3 selected years and considered RD-only patients with at least a readmission within 365 days of discharge. Patients readmitted over 365 days and outpatients were excluded from the analyses. The full-scale IQ was assessed using Wechsler Intelligence Scales [44].

In particular, Wechsler Intelligence Scale for Children–IV (WISC-IV) for school-aged children 6–16 and Wechsler Adult Intelligence Scale–IV (WAIS-IV) for adolescents aged from 16 years to adulthood.

In the selected period, 944 accesses to our ward occurred for a total number of 678 patients: 523 (77.1%) were hospitalized for the first and only time, and 155 (22.8%) had multiple hospitalizations. We excluded inpatients with a diagnosis of neurodevelopmental disorders from the analyses, which entered the ward with scheduled hospitalization or were admitted to the ward for checks, and cases with insufficient information. Exclusion criteria were the presence of Autism Spectrum Disorder, Intellectual Disability, and genetic syndromes.

The final sample consisted of 224 cases (165 females and 59 males). See Table 1 for demographic information. RD patients were 108 children/adolescents (mean age 15.58 ± 1.5; mean IQ 96.41 ± 16.3) with at least two psychiatric hospitalizations over the 3 selected years. The median time to readmission was 75.5 days (mean 124.47, SD 116.3). The control group (non-RD) consisted of 116 children/adolescents (mean age 14.44 ± 1.7; mean IQ 98.06 ± 15.2).

### 2.2. Procedure

Data were extracted from the unit ward register, which contained the records for each access taken. Specifically, records on personal data (age, gender), familial history of psychiatric disorders (yes/no), adoption (yes/no), substance use investigated through the toxicological analysis (yes/no), the presence of non-suicidal self-injurious (yes/no) and suicidal attempts (yes/no), as assessed by clinical examinations, length of stay (in days), post-discharge management (parental home vs. residential facility), the time elapsed between the first and the second admission (in days) were extracted and considered in the analyses. 

Inpatients at the Child and Adolescents Neuropsychiatry Unit ward underwent a child psychiatric examination conducted by experienced developmental psychiatrists and neuropsychologists. 

Psychiatric diagnoses were based on developmental history, extensive clinical examination, and a semi-structured interview [45] conducted with parent and patient, separately. Five groups of psychiatric diagnosis were identified as follow: Mood Disorder (59.8%, including, Depressive Disorders and Bipolar Disorders), Behavioural Disorder (16.07%, including Attention Deficit Hyperactivity Disorder (ADHD), Conduct Disorder, and Oppositional Defiant Disorder), Schizophrenic Disorder (12.9%, including Psychosis and Ultra-High Risk for Psychosis), Anxiety Disorders (5.8%), and Eating Disorders (5.3%, Anorexia, and Bulimia). Moreover, the number di patients who received a comorbid psychiatric diagnosis were considered (see Table 1).

**Table 1:** Demographic information of Revolving Door and non-Revolving Door patients.

	RD Patients *n*	Non-RD Patients *n*
Gender		
Male	23	36
Female	85	80
		
Age		
Pre-adolescents (≤14 years old)	32	44
Adolescents (>14 years old)	76	72
		
Diagnosis		
Mood Disorder	67	67
Behavioural Disorder	19	17
Schizophrenic Disorder	13	16
Eating Disorder	6	6
Anxiety Disorder	3	10
		
Comorbid Psychiatric Diagnosis		
Mood Disorder	7	5
Behavioural Disorder	10	16
Schizophrenic Disorder	1	2
Eating Disorder	1	2
Anxiety Disorder	8	5
		
Substance Use		
Cannabis	20	19
Cocaine	14	4
Benzodiazepine	8	6
Amphetamine	5	1
		
Self-injurious Attempt Behaviours		
Non-suicidal self-injurious	55	24
Suicidal attempts	32	30
		
Familial history of Psychiatric Disorders	61	56
		
Adopted child	38	6
		
Post Discharge Management		
Parental Home	79	101
Residential Facilities	29	15

RD = Revolving Door.

### 2.3. Instruments

The Kiddie Schedule for Affective Disorders and Schizophrenia Present and Lifetime Version (K-SADS-PL DSM-5) [45] is a semi-interview to determine the presence of past (lifetime) and current episodes of psychopathology in children and adolescents according to the DSM-5 criteria. The semi-interview is administered to children and their parents separately. The majority of items are scored using a 0 to 3 point rating scale. Scores of 0 indicate no information is available; scores of 1 suggest the symptom is not present; scores of 2 indicated sub-threshold presentation, and scores of 3 indicated threshold presentation of symptoms. 

The Children Global Assessment Scale (C-GAS) [46] is used to evaluate the level of impairment of the person’s overall functioning. Scores of 100–60 indicate adequate functioning, scores 60–40 the presence of evident problems, scores 40–30 the presence of serious problems, and scores below 30 serious/extreme compromises. 

Behavioural and emotional symptoms were assessed by the Achenbach System of Empirically Based Assessment (ASEBA) questionnaire. The Child Behaviour Check List Youth Self Report Forms 11–18 (CBCL-YSR) [38] is a well-known tool for detecting behavioural and emotional symptoms in children and adolescents. Youth are required to evaluate their own behaviours and emotions during the preceding 6 months on a 3-point Likert scale for each item (0 = Not True; 1 = Somewhat or Sometimes True; 2 = Very True or Often True). The hierarchical structure of the CBCL encompasses 113 items and several scales, as follows: (1) Syndrome Scales (Anxious/Depressed, Withdrawn/Depressed, Somatic Complaints, Social Problems, Thought Problems, Attention Problems, Rule-Breaking Behaviour, and Aggressive Behaviour); (2) Broad Band Scales (Internalizing Problems which incorporates Anxious/Depressed, Withdrawn/Depressed, Somatic Complaints; Externalizing Problems which incorporates Rule-Breaking Behaviour, and Aggressive Behaviour; Total Problems); (3) DSM-Oriented Scales (Affective Problems, Anxiety Problems, Somatic Problems, ADHD Problems, Oppositional Defiant Problems, Conduct Problems); and (4) 2007-Other Scales (Sluggish Cognitive Tempo, Obsessive-Compulsive Problems, Post-traumatic Stress Problems). According to the cut-off thresholds of Achenbach and Rescorla (2001), t-scores >69 are classified as clinically relevant, t-scores of 65–69 as borderline, and t-scores <65 as non-clinical symptoms. We analysed only t-scores of DSM-Oriented Scales (Affective Problems, Anxiety Problems, Somatic Problems, ADHD Problems, Oppositional Defiant Problems, Conduct Problems) because there were no overlapping items across the subscales.

The CBCL-YSR Dysregulation Profile (DP), characterized by simultaneous high values (above two standard deviations) in three syndrome scales (Anxious/Depressed, Attention Problems, Rule-Breaking Behaviour, and Aggressive Behaviour), was also calculated using the sum of t-scores of the three syndrome scales. Scores ≥210 are considered clinical, 180–209 in the borderline range, and ≤179 not-clinical. 

The presence of suicidal risk was assessed through the Columbia-Suicide Severity Rating Scale (C-SSRS) [43]. The trained psychiatrist asked questions to the patients to identify if the patient is at risk of suicide, assess the severity/immediacy of that risk, and the level of support that the person needs. The patients were asked if and when the thought of suicide (ideation) occurred, detailed information about specific plans for suicide, any steps taken toward enacting those plans, and if the individual was interrupted (by an outside circumstance) from starting the potentially self-injurious act. The questioner marks “yes” or “no,” as well as how recently the thought or behaviour occurred and the scoring of its severity. The shortest screeners are condensed to a minimum of two and a maximum of five questions. In this way, it is possible to obtain a score that goes from 0 to 5, where the score 5 indicates an increased suicide risk.

### 2.4. Statistical Analyses

The Shapiro–Wilk test was used to test the normality of the data and Levene’s test for the homogeneity of variances. Parametric tests were computed when data were normally distributed, and the assumption of homogeneity was not violated (see Results). When one assumption was not met, corrections were developed to produce a more valid critical value. 

For categorical variables, nonparametric analyses were conducted. Specifically, chi-square analyses were run to test differences between RD group and non-RD group on gender, presence of a familial history of psychiatric disorders, psychiatric diagnosis, psychiatric comorbidities, adoption, substances use, drug substance use, non-suicidal self-injurious suicidal attempts, and post-discharge management; Spearman’s correlations were run between the number of accesses and the time elapsed between the first and the second admission of RD group. 

For continuous variables, Student’s t-tests were used to compare RD and non-RD groups on age, IQ, length of stay, C-GAS, C-SSRS, DP, and the number of accesses. To correct for multiple testing, Bonferroni’s correction was used (*p*-value 0.05/7 < 0.007, after Bonferroni’s correction).

Multivariate Analysis of Variance (MANOVA) was used to compare RD and non-RD groups on 6 CBCL-YSR DSM-Oriented Scales (with the 6 CBCL-YSR DSM-Oriented Scales as dependent variables and groups as independent variable). Since the MANOVA assumption of homogeneity of variance was violated, Pillai’s Trace test was considered as the best option for its robustness. Post-hoc analyses were conducted by using Tukey’s HSD test (see Table 2). 

Pearson’s correlations were carried out to analyse the relationship between continuous variables (between the length of stay and DP scores; between the length of stay and C-GAS scores; between C-GAS scores and DP scores; between C-SSRS scores and DP scores).

Significant variables of rehospitalization were included in two separated models of logistic regression to address whether those variables increased the odd of RD. The regression models were presented as unadjusted and adjusted for age (pre-adolescents: ≤14 years old; adolescents: >14 years old), gender (females/males), and psychiatric diagnosis (Mood Disorder, Behavioural Disorder, Schizophrenic Disorder, Anxiety Disorders, Eating Disorders) because they may affect the predictive variables.

Numerical values (length of stay and age) were converted to categorical values in regression models. Moderation effects were examined by using multicollinearity tests for interaction. 

In the first model, step 1 included age, sex, and the psychiatric diagnosis as covariates. The previously identified categorical predictors (adoption, non-suicidal self-injurious, length of stay, post-discharge management) were entered in step 2 (see Table 3 for reference categories). In the second model, step 1 included age, sex, and the psychiatric diagnosis as covariates. The previously identified continuous predictors (C-SSRS, C-GAS, Affective Problems, Anxiety Problems, ADHD Problems, Oppositional Defiant Problems, Conduct Problems and DP) were entered in step 2.

The statistical software SPSS Version 22 (IBM Corporation) was used for analyses. 

## 3. Results

Out of the total sample, 51.8% of patients were hospitalized for the first and only time while 48.2% had multiple hospitalizations. RD and non-RD patients did not differ for age (t_222_ = 0.61, p = 0.54; Cohen’s d = 0.08), IQ (t_207_ = −0.75, *p* = 0.45; Cohen’s d = 0.10), gender (χ^2^_1_ = 2.73, *p* = 0.09; Φ = 0.11), familial history of psychiatric disorders (χ^2^_1_ = 1.5, *p* = 0.21; Φ = −0.82), psychiatric diagnosis (χ^2^_4_ = 3.91, *p* = 0.41; Cramer’s V = 0.13), and psychiatric comorbidities (χ^2^_4_ = 2.97, *p* = 0.57; Cramer’s V = 0.22). Considering adoption, the number of adopted children and adolescents was significantly higher in RD group than in non-RD group (χ^2^_1_ = 31.9, *p* < 0.001; Φ = 0.37). Out of 224 patients, 21.4% (48 patients) was positive for toxicological assays and 7.5% (17 patients) presented polysubstance use. RD and non-RD patients did not differ for substances use (χ^2^_1_ = 2.5, *p* = 0.11; Φ = −0.10) nor for drug substance use (χ^2^_3_ = 5.02, *p* = 0.17; Cramer’s V = −0.04). 

Considering readmitted patients, the rate of rehospitalization within three months was 85.1%, and after three months was 14.9%. Regarding the length of stay in days, the two groups differed significant (t_222_ = 3.1, *p* = 0.002; Cohen’s d = 0.35), with longer stay in RD group (mean 7.96, SD 4.3) than in non-RD group (mean 6.46, SD 4.2). A negative correlation was found in the RD group between the number of accesses and the number of days between the first and the second admission (more time elapsed for less number of accesses, Spearman’s r = −0.259, *p* = 0.007). 

However, considering C-GAS scores (see Table 2), RD group presented a greater functional impairment than non-RD group (t_222_ = −5.1, *p* < 0.0001; Cohen’s d = 0.68). A negative correlation (Pearson’s r = −0.209, *p* < 0.002) between C-GAS scores and length of stay was also found (lower C-GAS score for more days of hospitalization). C-GAS scores negatively correlated also with DP scores (lower C-GAS scores for higher DP scores, Pearson’s = −0.140, *p* = 0.037).

The presence of non-suicidal self-injurious was found in 35.27% of the whole group, in the 50.93% of RD group, and 20.69% of the non-RD group, with a significantly higher percentage in the RD group than in the non-RD group (χ^2^_1_ = 22.39, *p* < 0.0001; Φ = −0.36).

The presence of suicidal attempts was found in 27.6% of the whole group, in 29.63% of the RD group, and in 25.86% of the non-RD group, with a similar percentage in the RD group and the non-RD group (χ^2^_1_ = 0.45, *p* = 0.501; Φ = 0.04). Considering C-SSRS scores (see Table 2), RD patients were more at risk for suicide than non-RD patients (t_222_ = 4.2, *p* < 0.0001; Cohen’s d = 0.39). Moreover, we found a positive correlation between C-SSRS scores and DP scores (higher C-SSRS scores for higher DP scores, r = 0.318, *p* < 0.0001).

For CBCL-YSR DSM-Oriented Scales, a group effect was found (Pillai’s trace F_6,215_ = 5.58, *p* < 0.0001; η^2^_p_ = 0.13), with higher mean scores of RD group than non-RD group. From Tukey HSD post-hoc test (see Table 2), higher scores for the RD group than non-RD group were found in the following CBCL-YSR DSM-Oriented Scales: Affective Problems (*p* < 0.0001), Anxiety Problems (*p* = 0.031), ADHD Problems, (*p <* 0.0001), Oppositional Defiant Problems (*p* = 0.001) and Conduct Problems (*p* < 0.0001). No difference was found in Somatic Problems (*p* = 0.23). DP also differed between groups (t_201_ = 5.44, *p* < 0.0001; Cohen’s d = 0.11), with higher scores obtained in RD group than in non-RD group. Indeed, 44.4% of RD patients presented clinical DP scores (≥210), 35.1% borderline DP scores (between 180 and 209), and only 20.3% clinical scores (≤179). Conversely, 24.1% of non-RD patients showed clinical scores (≥210), 31.03% borderline scores (between 180 and 209), and 44.8% non-clinical scores (≤179). Moreover, it was found a positive correlation between length of stay and DP scores (more days of hospitalization for higher DP scores, Pearson’s r = 0.147, *p* = 0.028).

Regarding post-discharge management, RD patients were more likely to be placed in a residential facility than non-RD patients (χ^2^_1_ = 6.86, *p* = 0.009; Φ = 0.17). When the number of accesses was considered, patients placed in residential structures showed a greater number of accesses in the ward (t_222_ = 0.27, *p* = 0.001; Levene’s test t_47,640_ = 3.18, *p* = 0.003; Cohen’s d = 0.63) than patients lived at home (mean 3.02, SD 2.5 and mean 1.8, SD 1.1, respectively). Moreover, splitting post-discharge patients in two subgroups according to the post-discharge management (placed at home vs. placed in residential facility) and comparing them on C-GAS scores, patients placed in residential facility presented greater impairment in functioning than patients placed at home (t_222_ = −3.181, *p* = 0.002; Cohen’s d = 0.49). 

**Table 2:** Comparisons between Revolving Door and Non-Revolving Door patients on global functioning, suicidal risk, and behavioural and emotional symptoms.

	RD Patients Mean (SD)	Non-RD Patients Mean (SD)	*p*-Value
C-GAS	41.2 (8.8)	46.9 (8.0)	<0.0001
C-SSRS	3.7 (2.3)	2.4 (2.4)	<0.0001
			
CBCL-YSR Affective Problems	75.5 (12.02)	68.6 (11.8)	<0.0001
CBCL-YSR Anxiety Problems	64.2 (9.7)	61.4 (9.4)	0.031
CBCL-YSR ADHD Problems	61.6 (8.9)	57.4 (6.6)	<0.0001
CBCL-YSR Oppositional Defiant Problems	62.3 (9.9)	58.4 (7.3)	0.001
CBCL-YSR Conduct Problems	63.03 (11.9)	57.7 (8.8)	<0.0001
CBCL-YSR DP	206.1 (29.3)	186.4 (24.6)	<0.0001

RD = Revolving Door; C-GAS = Children Global Assessment Scale; C-SSRS = Columbia-Suicide Severity Rating Scale; CBCL-YSR = Child Behaviour Check List Youth Self Report Forms 11–18; DP = Dysregulation Profile.

Concerning the first logistic regression model, no interaction effect was found between any of the predictive variables. Having non-suicidal self-injurious and being adopted increased the odds of rehospitalizations (see Table 3). It should be noted that age, gender, and psychiatric diagnosis were not significant contributors to RD, but were considered for the adjusted model because they may have affected the predictive variables [9,16,22,24]. Even though the model was adjusted, no confounding effect was found between the predictive variables and age, gender, and psychiatric diagnosis.

Concerning the second logistic regression model, no interaction effect was found between any of the predictive variables. Having a greater functional impairment (C-GAS), suicidal risk (C-SSRS), and DP increased the odds of rehospitalizations (see Table 4). Moreover, in this logistic model, age, gender, and psychiatric diagnosis were not found to be significant contributors to RD but were considered for the adjusted model [9,16,22,24]. Even though the model was adjusted, no confounding effect was found between the predictive variables and age, gender, and psychiatric diagnosis.

## 4. Discussion

The current study aimed to better understand RD phenomenon considering multiple factors that are potentially associated with readmissions. 

Considering readmitted patients, the rate of rehospitalization within three months was 85.1%, and after three months was 14.9%. These rates were consistent with previous evidence showing that most readmissions occur 30–90 days after discharge [4,5].

We also found that as the number of days from the first discharge to readmission increased, the number of readmissions decreased. This result was probably related to the symptomatology of the patients: those presenting acute crisis required closer hospitalizations for symptoms stabilization [10], while patients discharged for longer times needed less intensive care and were likely less a threat to self or others [47]. Moreover, we found that RD patients had longer stay than non-RD patients.

Analysing the clinical conditions, our results documented that non-suicidal self-injurious were higher in RD than in non-RD patients and that these behaviours predict RD phenomenon. Self-injurious attempt behaviours have been already indicated as risk factors for rehospitalizations [14] and considered risk factors for future suicide attempts in psychiatric inpatients [48]. Moreover, we found a moderate positive correlation between the suicidal ideation severity scores (C-SSRS) and the number of readmissions of RD patients confirms previous findings [21,22,26], showing that RD adolescents were at greater risk for suicide than non-RD patients. The chronic nature of suicide risk [28], as well as the limited efficacy of interventions to prevent severe suicidal ideation and suicide attempts [49,50], could explain our and existing results in which severity of suicidal ideation has been related to rehospitalization [22,29,51]. 

We also found that the high presence of behavioural and emotional symptoms were associated with RD phenomenon. Indeed, results on CBCL-YSR documented higher scores in RD than in non-RD patients on DP and regarding CBCL-YSR DSM-Oriented Scales, on Affective Problems, Anxiety Problems, ADHD Problems, Oppositional Defiant Problems and Conduct Problems subscales. Moreover, our results showed that DP predicted repeated rehospitalizations and that the percentage of RD patients with clinical DP scores (44.4%) was higher than non-RD patients (24.1%). However, after controlling for age, gender, and psychiatric diagnosis, the odds of RD did not change but the significance of DP to predict rehospitalizations reduced (*p*-value of 0.08).

Furthermore, DP scores had a significantly low correlation with C-GAS and a significant moderate correlation with C-SSRS scores and length of stay. Altogether, these findings were in line with previous studies showing that patients with DP exhibit difficulties in regulating adverse states, elevated functional impairment [21,41,52,53], and were at high risk for psychopathologies, such as anxiety disorders, mood disorders, disruptive behaviours, substance use disorder, personality disorders, and suicide [39,40,41,42,54,55,56,57]. Our results showed the importance of recognizing emotional dysregulation during hospitalization to prevent further hospitalizations and adverse outcomes. 

Concerning functional impairment, we found that RD patients showed greater impairment than non-RD patients, as shown by lower C-GAS scores. The greater functional impairment in RD patients can be explained by multiple factors, including natural course of illness, medication non-adherence and non-efficacy, and inadequacy of out-of-hospital services [8]. Our results were consistent with previous findings, indicating that adolescents with higher functional impairment were more likely to experience frequent hospital admissions and be readmitted within a short period [5,58]. We also showed that RD patients with greater functional impairment were more likely to be placed in residential facilities, in line with evidence that associated greater functional impairment to more post-discharge intensive interventions [58]. Accordingly, adolescent patients placed in restrictive assistance outside their home were mainly those with greater cognitive, psychiatric, and behavioural impairment [59] and were more vulnerable for repeated rehospitalizations [10]. The higher rate of readmission among children and adolescents living in residential facilities may also be due to the lower threshold adopted by residential treatment centres to send to hospitalization to minimize liability [2].

Regarding psychiatric diagnosis and comorbidities, our results showed that RD and non-RD patients did not differ for psychopathological disorders. So far, studies on the association between psychiatric diagnosis and repeated rehospitalizations have reported contradictory results. Some studies revealed that psychiatric diagnoses were not predictive for rehospitalization [8,12,13], while others [6,16,19,32] found that psychosis [15,16,17,18] and schizophrenic disorder [19,20] were at high risk for RD. Discrepancies between studies may be due to participants’ characteristics. For example, in our study, participants mainly received a diagnosis of mood disorder (59.8% of patients) and only 12.95% of schizophrenic disorder, while in the study by Cheng and colleagues [19] the participants presented more heterogeneous diagnoses (14.1% Substance Use Disorder, 13.2% Schizophrenic or Psychotic Disorder, 26.6%, Mood Disorder, 8.1% Anxiety Disorder, 2.4% Personality Disorder, 34.9% Other Disorder). These differences may be due to the age of participants, younger in our study (ages 6–18, mean 15 years old) and older (ages 18 to 24, mean 18 years old) in the study by Cheng and colleagues [19].

Within our patients, 21.4% was positive at the toxicological assays, but no difference between RD and non-RD patients was found in substance use. This result confirms previous findings showing no increase in readmission in adolescents with substance abuse [2,5,15]. 

Furthermore, we investigated the role of socio-demographic factors as age, gender, IQ, familial history of psychiatric disorders, and adoption for RD phenomenon. Results demonstrate adoption status as a risk factor for repeated rehospitalizations, thereby reinforcing a recent observation of Edgcomb and colleagues [58]. 

Moreover, several factors such as social circumstances prior to adoption, the background of the adoptive family, and relationships between the adopted child and his/her family members, in addition to the genetic and familial history of psychiatric disorders, may exacerbate developmental difficulties and psychiatric symptoms. It has been observed that adopted children have high rates of mental health disorders [60,61], with a predominance of externalizing disorders [62]. 

Regarding the familial history of psychiatric disorders, we did not find a specific association with repeated rehospitalizations. Albeit family history of mental illness may increase the risk for the same disorders in the next generations [34], previous findings, according to ours, showed a family history of psychiatric diseases is not predictive for multiple hospitalizations [11,13,14]. 

Moreover, we did not find differences in age between RD and non-RD groups. Existing studies reported contradictory results: some failed to find a relation between age and RD [24,32], while others documented younger [12,15,16,63,64], as well as older adolescents [2,5,19], were more likely to be readmitted to the psychiatric ward. Differences in participant numerosity as well as in age and diagnosis may explain the divergence in results.

## 5. Conclusions

In the present study, clinical instruments and different factors affecting readmissions are put together to provide a more comprehensive picture of RD phenomenon.

We found that more than half of psychiatric patients are readmitted from discharge within three months, RD patients have a history of adoption, need longer stays, and are more frequently placed in residential facilities than non-RD patients. 

We also found that clinical aspects and measures, such as high functional impairment, non-suicidal self-injurious, suicidal ideation severity, behavioural and emotional symptoms, and emotional dysregulation, are associated with rehospitalization. 

Early attention to these aspects can help clinicians prevent further hospitalizations and improve mental health care services.

## Figures and Tables

**Table 3:** First logistic regression model predicting Revolving Door with the reference category for each variable considered.

	Odds Ratio	95% CI	*p*-Value	R^2^
Unadjusted model				0.23
Adopted child	6.65	2.55–17.31	<0.0001	
Non-suicidal self-injurious	4.12	2.14–7.94	<0.0001	
Residential facilities	0.78	0.34–1.76	0.551	
Length of stay	1.73	0.89–3.37	0.106	
				
Adjusted model				0.23
Adopted child	6.93	2.59–18.53	<0.0001	
Non-suicidal self-injurious	4.06	2.09–7.86	<0.0001	
Residential facilities	0.76	0.33–1.75	1.75	
Length of stay	1.63	0.82–3.23	0.159	

**Table 4:** Second logistic regression model predicting Revolving Door with the reference category for each variable considered.

	Odds Ratio	95% CI	*p*-Value	R^2^
Unadjusted model				0.24
C-GAS	1.10	1.04–1.14	<0.0001	
C-SSRS	0.83	0.72–0.95	0.009	
CBCL-YSR Affective Problems	1.00	0.96–1.04	0.96	
CBCL-YSR Anxiety Problems	1.04	0.99–0.93	0.06	
CBCL-YSR ADHD Problems	0.99	0.93–1.05	0.77	
CBCL-YSR Oppositional Defiant Problems	1.01	0.96–1.06	0.58	
CBCL-YSR Conduct Problems	0.98	0.94–1.02	0.34	
CBCL-YSR DP	0.97	0.94–0.99	0.03	
				
Adjusted model				0.25
C-GAS	1.10	1.05–1.15	<0.0001	
C-SSRS	0.84	0.72–0.98	0.02	
CBCL-YSR Affective Problems	0.99	0.95–1.04	0.37	
CBCL-YSR Anxiety Problems	1.02	0.97–1.07	0.28	
CBCL-YSR ADHD Problems	0.99	0.92–1.05	0.8	
CBCL-YSR Oppositional Defiant Problems	1.01	96–1.06	0.49	
CBCL-YSR Conduct Problems	0.97	0.94–1.01	0.27	
CBCL-YSR DP	0.98	0.97–1.00	0.10	

## Data Availability

The data presented in this study are available on request from the corresponding author. The data are not publicly available due to privacy and ethical restrictions.
